# Preoperative risk stratification for pathological upgrading in colorectal polyps using explainable machine learning: implications for screening optimization and resource allocation

**DOI:** 10.3389/fpubh.2026.1845071

**Published:** 2026-05-21

**Authors:** Bo Yang, Chang Zhang, Mingsu Gong

**Affiliations:** 1 Department of Gastroenterology and Hepatology, Guizhou Aerospace Hospital, Zunyi, China; 2 Department of Gastroenterology, The Fourth Affiliated Hospital of Anhui Medical University, Hefei, China; 3 Anhui Medical University, Hefei, China

**Keywords:** colorectal cancer prevention, colorectal polyps, Explainable artificial intelligence (SHAP), machine learning, risk stratification

## Abstract

**Background:**

Pathological upgrading in colorectal polyps, which occurs when resection specimens have a higher histological grade than preoperative biopsies, can lead to an underestimation of the severity of the disease and suboptimal treatment. Given the substantial global burden of colorectal cancer (CRC), improving preoperative risk stratification of colorectal polyps is essential for optimizing screening strategies and resource allocation in CRC prevention.

**Methods:**

Between December 2019 and December 2024, 593 patients with colorectal polyps were included in this retrospective study, undergoing endoscopic biopsy and subsequent complete resection. Clinical and endoscopic variables were gathered, and feature selection was conducted using LASSO regression and the Boruta algorithm. Repeated ten-fold cross-validation was used to develop and evaluate six machine learning models: RF, CART, NNet, LR, GBM, and XGBoost. AUC, accuracy, sensitivity, specificity, MCC, and Brier score were used to assess the model's performance. A web-based calculator was developed to aid in clinical implementation, using SHapley Additive exPlanations (SHAP) to interpret the optimal model.

**Results:**

Among the 593 patients, 150 (25.3%) experienced pathological upgrading. Five key predictors were identified: maximum tumor diameter, surface color, erosion, villous structure, and lesion location. With an AUC of 0.890 in the training set and 0.863 in the test set, the XGBoost model exhibited superior performance, along with strong calibration and discrimination. SHAP analysis showed that lesion location, particularly rectal location, was the most influential factor, followed by erosion and tumor size. To aid in individual risk prediction, a user-friendly online calculator was designed (available at: https://changchangzhang2001.shinyapps.io/blsj/). The calculator will remain freely accessible for at least 3 years after publication, and the source code is available from the corresponding author upon reasonable request.

**Conclusion:**

Researchers in this study devised a machine learning model that provides explanations for predicting pathological upgrading in colorectal polyps. The model serves as a useful instrument for assessing preoperative risks and aids in making decisions based on risk, potentially boosting early detection efficiency, refining endoscopic management approaches, and improving resource allocation in colorectal cancer prevention programs.

## 1 Introduction

Recognized as key precancerous lesions, colorectal polyps are linked to colorectal cancer (CRC), a significant public health problem worldwide (1, 2). Despite improvements in screening programs, CRC remains a prevalent malignancy globally, significantly impacting morbidity, mortality, and healthcare resources. Evidence has shown that colonoscopy screening and early removal of colorectal polyps can effectively interrupt the progression to high-grade dysplasia or invasive cancer, thereby reducing the overall burden of CRC (3–5). Therefore, optimizing the detection, risk assessment, and management of colorectal polyps is of critical importance not only for individual patient outcomes but also for improving the efficiency of CRC prevention strategies at the population level.

Colorectal polyp detection rates have notably increased due to the widespread adoption of colonoscopy. However, a persistent challenge in clinical practice is the discrepancy between preoperative endoscopic biopsy findings and the final pathological results after complete resection (6–8). Such inconsistencies are particularly common in large or morphologically complex lesions and may lead to underestimation of disease severity, delayed intervention, or inappropriate treatment strategies. From a public health perspective, inaccurate risk assessment may result in both undertreatment of high-risk lesions and overuse of medical resources in low-risk cases, thereby reducing the efficiency of screening programs. Although strategies such as the “resect and discard” approach have been proposed to improve efficiency, concerns regarding diagnostic variability, technical limitations, and medico-legal risks have limited their widespread adoption. Consequently, there remains a critical need for reliable tools that can support accurate preoperative risk stratification and guide resource allocation in CRC prevention.

Accurately identifying polyps at high risk of pathological upgrading remains challenging. Traditional clinical indicators, such as polyp size, number, and morphology, are commonly used for risk assessment; however, their predictive performance is limited and often subjective. Furthermore, conventional statistical models are restricted by assumptions of linear relationships and have a limited ability to capture complex interactions among variables, potentially reducing their effectiveness in real-world clinical applications (9). In contrast, machine learning approaches offer advantages in dealing with high-dimensional data, modeling non-linear relationships, and integrating heterogeneous clinical and endoscopic features, making them promising tools for improving risk stratification and supporting data-driven decision-making in large-scale screening and prevention programs (10, 11).

Despite these advantages, the lack of interpretability remains a major barrier to the clinical and public health implementation of machine learning models. Clinicians and healthcare systems require transparent and explainable tools to ensure trust, facilitate decision-making, and enable integration into routine practice. Explainable AI methods like SHapley Additive exPlanations (SHAP) enhance model interpretability and practical use by quantifying each feature's contribution to predictions.

Thus, this research focused on pinpointing major risk factors linked to pathological upgrading in colorectal polyps and on creating and validating a prediction model based on machine learning, utilizing commonly available clinical and endoscopic features. Furthermore, we incorporated SHAP to improve model interpretability and developed an online calculator to facilitate real-world application. By enabling accurate preoperative risk stratification, this study seeks to support risk-based decision-making, optimize endoscopic management strategies, and improve the efficiency of colorectal cancer prevention and screening programs at the population level.

## 2 Methods

### 2.1 Study design and population

This research collected clinical data from patients who were hospitalized at the Fourth Affiliated Hospital of Anhui Medical University between December 2019 and December 2024. All patients underwent colonoscopy, were found to have colorectal polyps, and received pathological biopsies. Data collection was done through the hospital's electronic medical record system. Every procedure utilized a high-definition endoscopy system (Olympus EVIS X1 with a CF-HQ190L colonoscope), focusing on white-light imaging for lesion assessment.

Inclusion criteria: (1) All patients underwent endoscopic biopsy prior to surgery. Biopsy-confirmed adenomatous polyps were then completely removed by endoscopic resection, and all post-operative specimens were sent for pathological examination. (2) Complete clinical data and blood test results were available for all patients before they received their first treatment. (3) It was verified that the lesions were the same based on the features described in the endoscopic forceps biopsy (EFB) and endoscopic submucosal dissection (ESD) or endoscopic mucosal resection (EMR) operation reports. (4) There was no history of other primary tumors, and no history of familial adenoma or colon cancer. (5) The time interval between biopsy and EMR or ESD was < 2 months.

Exclusion criteria: (1) Lesions that were removed endoscopically without prior biopsy, or those with biopsy results indicating non-neoplastic polyps (such as hyperplastic or inflammatory polyps), were excluded from the study. (2) Patients with diseases that seriously affected examination, treatment, or follow-up were excluded, such as coagulation disorders, severe liver, kidney, or heart diseases, and mental disorders. (3) Patients without complete follow-up data were excluded. (4) Patients were not included if there was no confirmation that the biopsy and resection specimens were from the identical lesion. (5) Patients with a previous diagnosis of gastrointestinal malignant tumors were excluded. (6) The interval between biopsy and endoscopic resection exceeded 2 months. (7) Cases with deep submucosal invasion (≥1,000 μm) identified on postoperative pathology were excluded.

### 2.2 Outcome definition

Outcome measure: pathological upgrading of colorectal polyps was defined as the primary study endpoint. All resection specimens underwent independent review by two knowledgeable pathologists who were not informed of the biopsy findings. The 2019 5th edition of the World Health Organization's Classification of Tumors of the Digestive System was utilized for determining diagnoses. Any conflicts were resolved by a third senior pathologist. A pathological upgrade was defined as a higher histological grade in the postoperative specimen compared with the preoperative biopsy. This included cases in which adenomatous polyps identified on biopsy were upgraded to low-grade intraepithelial neoplasia (LGIN), high-grade intraepithelial neoplasia (HGIN), or adenocarcinoma after resection; LGIN was upgraded to HGIN or adenocarcinoma; and HGIN was upgraded to adenocarcinoma. Intramucosal carcinoma (Tis) and superficial submucosal invasion were included in the analysis, whereas cases with deep submucosal invasion (≥1,000 μm) were excluded, as such lesions are generally considered indications for surgical rather than endoscopic treatment. Therefore, the analyzed cohort primarily consisted of lesions suitable for endoscopic intervention. If the postoperative pathology results were consistent with the preoperative biopsy findings, the case was classified as “no upgrade.”

### 2.3 Variable definition and data extraction

Clinical and endoscopic variables were collected from hospital records and colonoscopy reports. Clinical variables included sex, age, body mass index (BMI), smoking history, family history of colorectal cancer, preoperative carcinoembryonic antigen (CEA) level, combined metabolic syndrome (CMS), and intestinal cleanliness. BMI was categorized as < 24, 24–28, and >28 kg/m^2^. CEA was classified as normal (< 4.7 μg/L) or elevated (≥4.7 μg/L). CMS referred to the presence of hypertension, diabetes, coronary heart disease, or hyperlipidemia. Endoscopic variables included the number of biopsy specimens, maximum tumor diameter (MTD), lesion morphology, number of lesions, surface characteristics, erosion or ulceration, villous structure, and lesion location. Morphology was classified as sessile or pedunculated, and the number of biopsy samples was grouped as 1 or ≥2. Surface color was recorded as either similar to normal mucosa or abnormal (congested, red, or rough). Lesion location was categorized as the ascending colon, transverse colon, descending colon, sigmoid colon, or rectum. Maximum tumor diameter (MTD) was dichotomized as < 10 mm and ≥10 mm. The 10-mm cutoff was selected for several reasons. First, this threshold is widely recognized in major clinical guidelines, including those from the European Society of Gastrointestinal Endoscopy (ESGE) and the US Multi-Society Task Force (USMSTF), as a criterion for defining advanced adenomas and is commonly used in risk stratification and surveillance recommendations (12, 13). Second, dichotomization facilitates rapid clinical assessment and supports the development of a simplified and user-friendly prediction tool, thereby enhancing practical applicability. Third, the use of this threshold allows consistency with prior studies, enabling comparability of findings (14). In this study, MTD was intentionally modeled as a binary variable to prioritize clinical interpretability and usability. Alternative approaches, such as modeling MTD as a continuous variable or using multiple categorical thresholds, were not explored and should be investigated in future studies. For patients with multiple polyps, only the largest lesion was included in the final analysis.

### 2.4 Statistical analysis

Missing values were handled using multiple imputation by chained equations (MICE) implemented in the “mice” package in R. Five imputed datasets were generated. Predictive mean matching (PMM) was used for continuous variables, logistic regression for binary variables, and multinomial regression for unordered categorical variables. All variables included in the analysis were incorporated into the imputation model to improve robustness. Final estimates were obtained by pooling results across the imputed datasets according to Rubin's rules. Continuous variables were summarized as medians with interquartile ranges (IQRs), whereas categorical variables were presented as frequencies and percentages. Between-group comparisons were conducted using the Mann–Whitney *U* test for continuous variables and either the chi-square test or Fisher's exact test for categorical variables, as appropriate. All eligible patients were randomly assigned to a training cohort and a test cohort in a 7:3 ratio. To identify robust candidate predictors while reducing redundancy among variables, feature selection was performed using both least absolute shrinkage and selection operator (LASSO) regression and the Boruta algorithm. The retained variables were further examined by correlation analysis, and their pairwise relationships were visualized using a correlation heatmap. Correlation coefficients >0.4 were considered potentially meaningful.

Based on the selected variables, six machine learning algorithms were developed, including classification and regression tree (CART), gradient boosting machine (GBM), logistic regression (LR), neural network (NNet), random forest (RF), and extreme gradient boosting (XGBoost). Model development and internal validation were performed using repeated ten-fold cross-validation. Discriminative performance was primarily assessed by the area under the receiver operating characteristic curve (AUC). To evaluate the statistical significance of differences in AUC among the six machine learning models, pairwise comparisons of AUCs in the test cohort were conducted using the DeLong test. Additional metrics, including accuracy, sensitivity, specificity, Kappa, and Matthews correlation coefficient (MCC), were also used to provide a broader evaluation of model performance (15–17). Model calibration was assessed using the Brier score and calibration plots (18), while decision curve analysis (DCA) was applied to estimate potential clinical utility across a range of threshold probabilities. Given the moderate degree of class imbalance, no resampling techniques (e.g., SMOTE or ROSE) or class weighting strategies were employed. The final model was selected on the basis of its overall discrimination, calibration, and classification performance. To improve interpretability, SHapley Additive exPlanations (SHAP) were used to quantify the contribution of each predictor at both the global and individual levels (19–21). In addition, a web-based calculator was constructed to facilitate practical application of the best-performing model (22–25). All analyses and model development procedures were conducted using R software (version 4.5.2).

## 3 Results

### 3.1 Analysis flowchart

**Figure 1:**
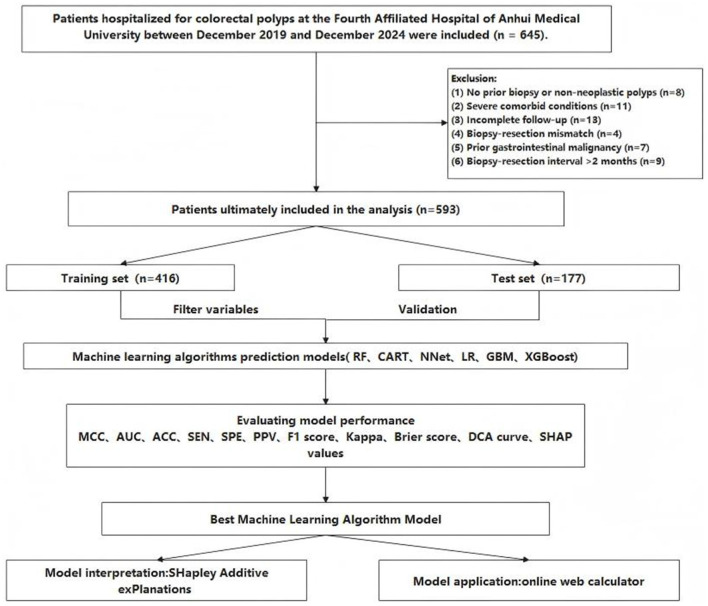
Analysis flowchart.

Once the inclusion and exclusion criteria were applied, 593 patients were included in the study, with 150 (25.3%) exhibiting pathological upgrading after undergoing resection. The extent of missing data for each variable is summarized in Supplementary Table S1, showing that the overall proportion of missing values was low. The process of screening patients and analyzing data is outlined in Figure 1. The complete dataset was randomly separated into a training group (70%, *n* = 416) and a test group (30%, *n* = 177). Supplementary Table S2 indicates that there were no notable differences between the two groups. Each patient had 28 variables collected in total. Table 1 summarizes the baseline characteristics of patients in the training group.

**Table 1:** Patient characteristics in the training set.

Variables	Total (*n* = 416)	Non-upgraded group (*n* = 311)	Upgraded group (*n* = 105)	*P*-value
**Gender [*****n*** **(%)]**				
Female	58 (13.9%)	44 (14.1%)	14 (13.3%)	0.964
Man	358 (86.1%)	267 (85.9%)	91 (86.7%)	
Age [*M* (Q1–Q3, years]	58 (51-72)	58 (51-71)	58 (50-73)	0.889
**Smoking history [*****n*** **(%)]**				
No	79 (19.0%)	60 (19.3%)	19 (18.1%)	0.899
Yes	337 (81.0%)	251 (80.7%)	86 (81.9%)	
**BMI [*****n*** **(%)]**				
24–28 kg/m^2^	210 (50.5%)	146 (46.9%)	64 (61.0%)	0.030
< 24 kg/m^2^	132 (31.7%)	103 (33.1%)	29 (27.6%)	
>28 kg/m^2^	74 (17.8%)	62 (19.9%)	12 (11.4%)	
**CEA [*****n*** **(%)]**				
< 4.7 μg/L	363 (87.3%)	269 (86.5%)	94 (89.5%)	0.525
≥4.7 μg/L	53 (12.7%)	42 (13.5%)	11 (10.5%)	
**Family history of CRC [*****n*** **(%)]**				
No	377 (90.6%)	285 (91.6%)	92 (87.6%)	0.304
Yes	39 (9.4%)	26 (8.4%)	13 (12.4%)	
**CMS [*****n*** **(%)]**				
No	268 (64.4%)	203 (65.3%)	65 (61.9%)	0.613
Yes	148 (35.6%)	108 (34.7%)	40 (38.1%)	
**Maximum tumor diameter [*****n*** **(%)]**				
< 10 mm	222 (53.4%)	192 (61.7%)	30 (28.6%)	< 0.001
≥10 mm	194 (46.6%)	119 (38.3%)	75 (71.4%)	
**Pedunculated tumor [*****n*** **(%)]**				
Sessile	165 (39.7%)	125 (40.2%)	40 (38.1%)	0.791
Pedunculated	251 (60.3%)	186 (59.8%)	65 (61.9%)	
**Number of biopsy blocks [*****n*** **(%)]**				
1 piece	342 (82.2%)	254 (81.7%)	88 (83.8%)	0.728
≥2 pieces	74 (17.8%)	57 (18.3%)	17 (16.2%)	
Villous				
No	221 (53.1%)	191 (61.4%)	30 (28.6%)	< 0.001
Yes	195 (46.9%)	120 (38.6%)	75 (71.4%)	
Surface [*n* (%)]				
Normal mucosal color	159 (38.2%)	143 (46.0%)	16 (15.2%)	< 0.001
Red	257 (61.8%)	168 (54.0%)	89 (84.8%)	
**Erosion [*****n*** **(%)]**				
No	241 (57.9%)	214 (68.8%)	27 (25.7%)	< 0.001
Yes	175 (42.1%)	97 (31.2%)	78 (74.3%)	
**Number of tumor [*****n*** **(%)]**				
Single	302 (72.6%)	226 (72.7%)	76 (72.4%)	1.000
Multiple	114 (27.4%)	85 (27.3%)	29 (27.6%)	
**Intestinal cleanliness [*****n*** **(%)]**				
Adequate bowel preparation	394 (94.7%)	298 (95.8%)	96 (91.4%)	0.137
Inadequate bowel preparation	22 (5.3%)	13 (4.2%)	9 (8.6%)	
**Location [*****n*** **(%)]**				
Ascending colon	48 (11.5%)	42 (13.5%)	6 (5.7%)	< 0.001
Transverse colon	48 (11.5%)	43 (13.8%)	5 (4.8%)	
Descending colon	70 (16.8%)	64 (20.6%)	6 (5.7%)	
Sigmoid colon	109 (26.2%)	102 (32.8%)	7 (6.7%)	
Rectum	141 (34.0%)	60 (19.3%)	81 (77.1%)	

### 3.2 Feature selection

**Figure 2:**
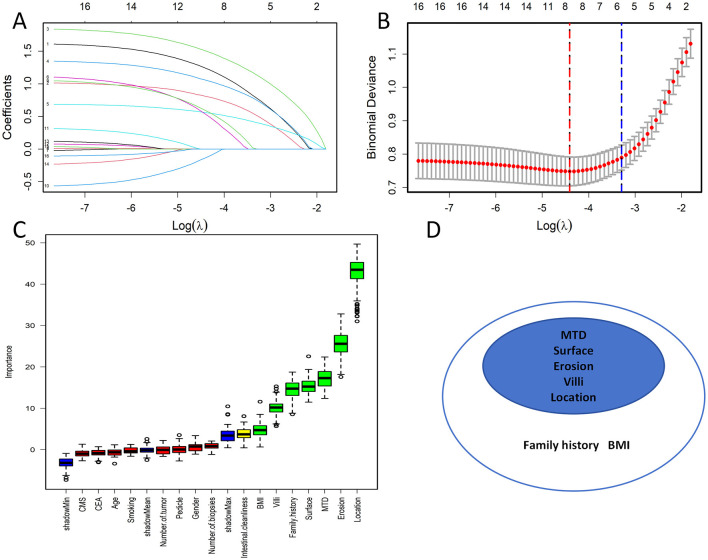
**(A)** Lasso coefficient path map. Each color curve represents a trend in variance coefficient change. **(B)** Lasso cross-validation curve.The red vertical line on the left represents λ min, while the blue vertical line on the right represents λ 1se. Here, λ min refers to the lambda value corresponding to the minimum mean squared error (MSE) among all tested values. In contrast, λ 1se denotes the lambda value for the simplest and best model obtained through cross-validation within one standard error of λ min. **(C)** Boruta Feature Selection. **(D)** Feature Intersection Plot.

To limit the effect of multicollinearity in model construction, LASSO regression was applied for variable screening. At the lambda.1se value of 0.0371793, five predictors with non-zero coefficients were retained. Feature selection was carried out using the Boruta algorithm to further affirm the importance of variables. By employing random forests, Boruta iteratively selects features by comparing them against shuffled “shadow features” that serve as a noise standard. To ensure statistically validated relevance, a feature is retained only if its permutation importance (*Z*-score) consistently outperforms the maximum shadow feature score during the iterative process. This approach gets rid of spurious correlations, boosts the stability of high-dimensional data, and successfully pinpoints robust yet subtle predictors (26, 27). The Boruta algorithm, through its iterative process, identified seven significant variables. Only features retained by both LASSO and Boruta were included in subsequent modeling. The shared subset of predictors consisted of maximum tumor diameter, polyp surface color, presence of erosion on the polyp, presence of villous structure, and polyp location, as illustrated in Figure 2.

### 3.3 Correlation analysis

**Figure 3:**
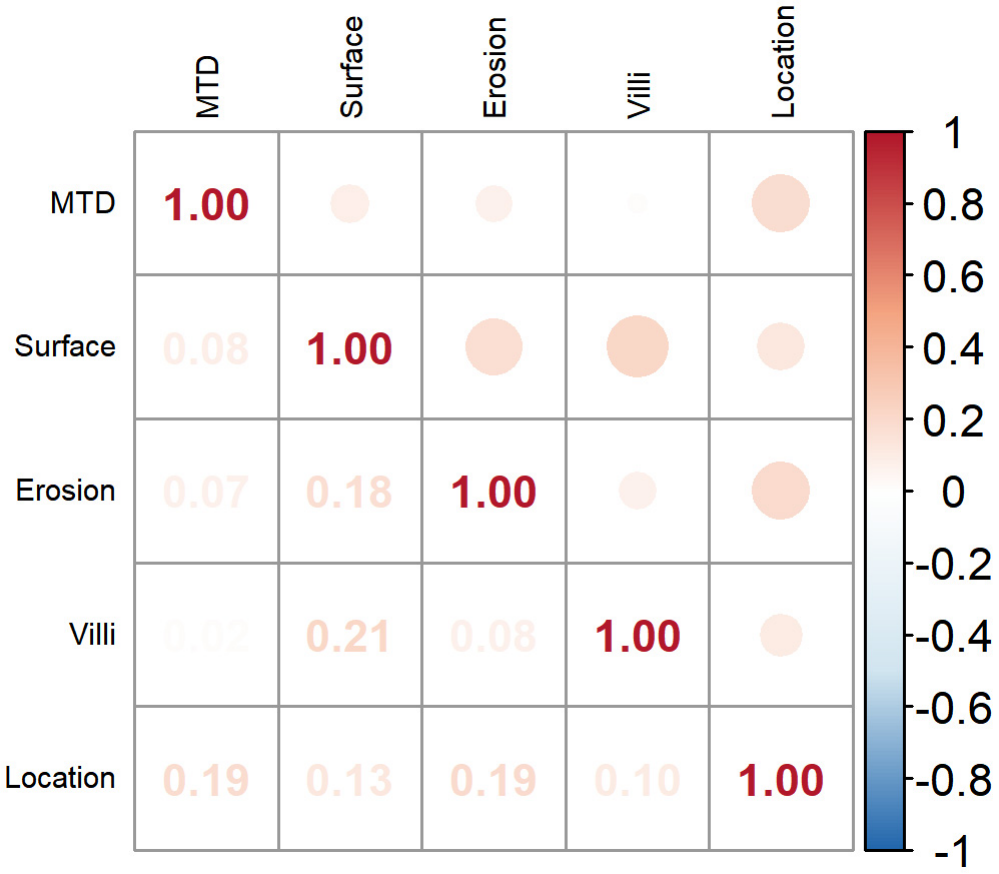
Heatmap of correlation analysis between variables.

To examine the associations among the five retained variables, a correlation analysis was carried out, and the results were presented in a heatmap (Figure 3). All pairwise correlation coefficients were below 0.4, suggesting that multicollinearity was not a concern in the selected variables. The input features derived from these variables were employed to create prediction models using six machine learning methods, namely RF, CART, NNet, LR, GBM, and XGBoost.

### 3.4 Multi-model performance evaluation

**Figure 4:**
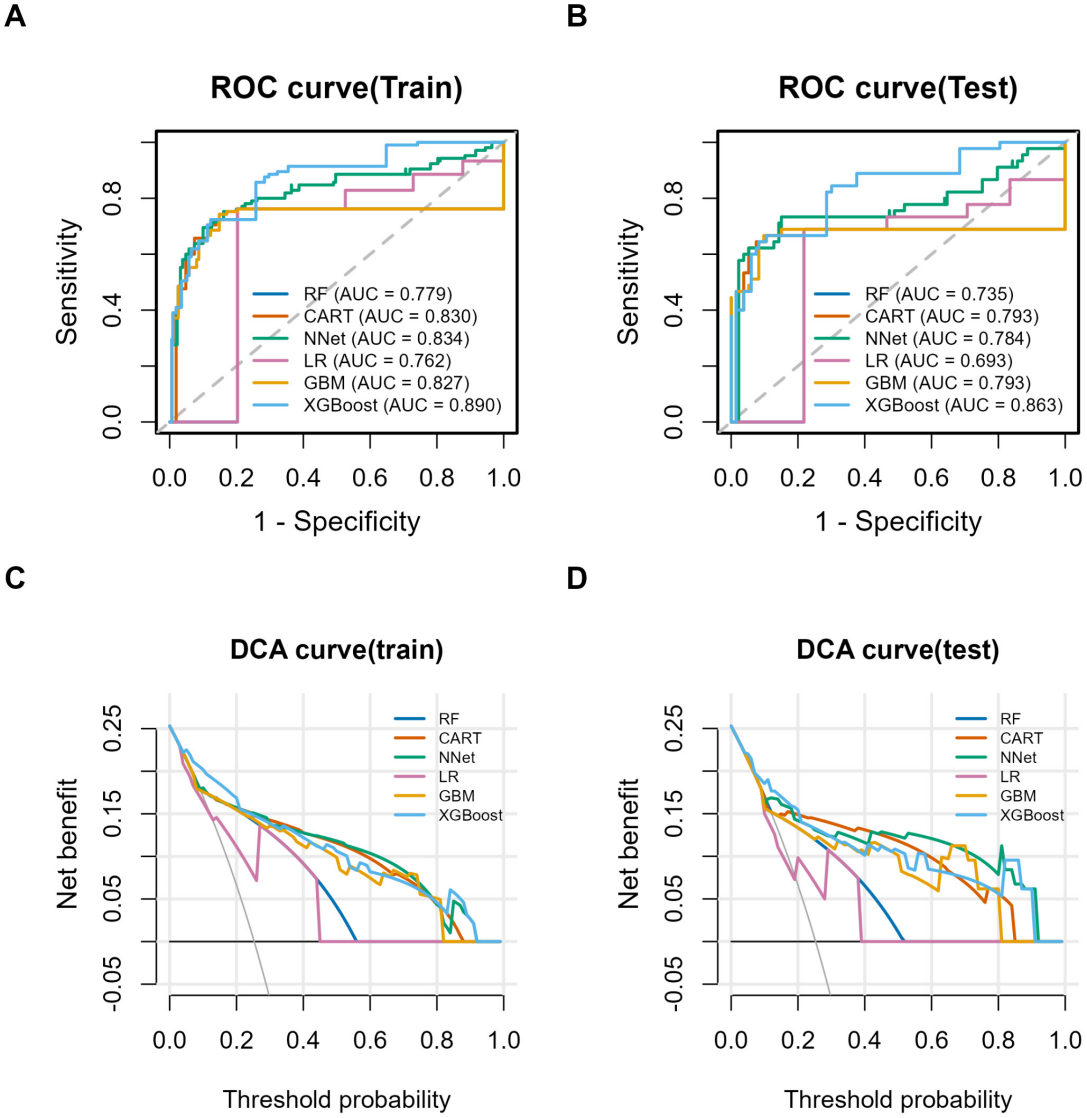
ROC curves and DCA curves for each model in the training and test sets. **(A)** ROC curve in the training set. **(B)** ROC curve in the test set. **(C)** DCA curve in the training set. **(D)** DCA curve in the test set.

The six machine learning models' predictive performance was assessed through ten-fold cross-validation, with ROC analysis indicating that the XGBoost model had the highest overall discriminative capability among the algorithms. In the training cohort, the AUCs (95% CIs) of RF, CART, NNet, LR, GBM, and XGBoost were 0.779 (0.732–0.826), 0.830 (0.781–0.878), 0.834 (0.780–0.888), 0.762 (0.705–0.817), 0.827 (0.778–0.876), and 0.890 (0.856–0.924), respectively. In the test cohort, the corresponding AUCs were 0.735 (0.658–0.812), 0.793 (0.710–0.875), 0.784 (0.685–0.882), 0.693 (0.593–0.791), 0.794 (0.711–0.875), and 0.863 (0.799–0.926), confirming the superior discrimination of XGBoost (Figures 4A, B). Notably, logistic regression, as a traditional statistical baseline model, showed lower discriminative performance (test AUC: 0.693) compared with the XGBoost model (0.863). In the test cohort, XGBoost achieved the highest AUC (0.863). Pairwise comparisons using the DeLong test showed that the AUC of the XGBoost model was significantly higher than those of all other models (all *P* < 0.05), indicating that its superior discriminative performance is unlikely to be attributable to random variation. Detailed results of the DeLong test are provided in Supplementary Table S3.

Additional performance metrics are summarized in Table 2. In the training set, XGBoost achieved an accuracy of 0.839, sensitivity of 0.724, specificity of 0.877, precision of 0.667, F1 score of 0.694, Kappa of 0.585, Brier score of 0.165, and MCC of 0.586. In the test set, the corresponding values were 0.815, 0.667, 0.865, 0.625, 0.645, 0.520, 0.170, and 0.520, respectively. Overall, the XGBoost model maintained stable performance across the training and test cohorts, indicating good generalizability. Among the remaining models, CART, NNet, and GBM also showed relatively favorable predictive performance, with test-set AUCs of 0.793, 0.784, and 0.794, respectively. Notably, CART achieved the highest Matthews correlation coefficient (MCC) in the test cohort (0.566), whereas the NNet model yielded the lowest Brier score (0.110), indicating superior calibration performance in terms of probability estimation. These findings suggest that although XGBoost demonstrated the best discriminative ability, NNet showed better agreement between predicted probabilities and observed outcomes. However, when discrimination, calibration, and classification metrics were considered collectively, this study prioritized discriminative performance (AUC) as the primary criterion for model selection. Based on its highest AUC in both the training and test cohorts, along with stable generalization performance, XGBoost was selected as the overall optimal model. Nevertheless, the relative advantage of NNet in calibration should be acknowledged.

**Table 2:** Evaluation metrics of the models constructed by each algorithm.

Dataset	Model	AUC	ACC	SEN	SPE	Precision	F1 score	Kappa	Brier score	MCC
Train	RF	0.779	0.788	0.762	0.797	0.559	0.645	0.499	0.178	0.511
	CART	0.830	0.841	0.705	0.887	0.679	0.692	0.585	0.111	0.585
	NNet	0.834	0.848	0.695	0.900	0.702	0.699	0.597	0.109	0.597
	LR	0.762	0.788	0.762	0.797	0.559	0.645	0.499	0.189	0.511
	GBM	0.827	0.824	0.743	0.852	0.629	0.681	0.561	0.148	0.565
	XGBoost	0.890	0.839	0.724	0.877	0.667	0.694	0.585	0.165	0.586
Test	RF	0.735	0.758	0.689	0.782	0.517	0.591	0.424	0.187	0.433
	CART	0.793	0.837	0.667	0.895	0.682	0.674	0.566	0.116	0.566
	NNet	0.784	0.815	0.644	0.872	0.630	0.637	0.513	0.110	0.513
	LR	0.693	0.758	0.689	0.782	0.517	0.591	0.424	0.189	0.433
	GBM	0.794	0.809	0.689	0.850	0.608	0.646	0.516	0.153	0.518
	XGBoost	0.863	0.815	0.667	0.865	0.625	0.645	0.520	0.170	0.520

Calibration curves were further used to visually assess agreement between predicted and observed probabilities. Calibration curves for all six models in both the training and test cohorts are presented in Supplementary Figure S1. Decision curve analysis demonstrated that all six models provided net clinical benefit across a wide range of threshold probabilities, as their curves remained above the “treat-all” and “treat-none” strategies (Figures 4C, D). Taken together, these findings support XGBoost as the optimal model for predicting pathological upgrading in colorectal polyps.

### 3.5 SHAP-based model interpretability analysis

**Figure 5:**
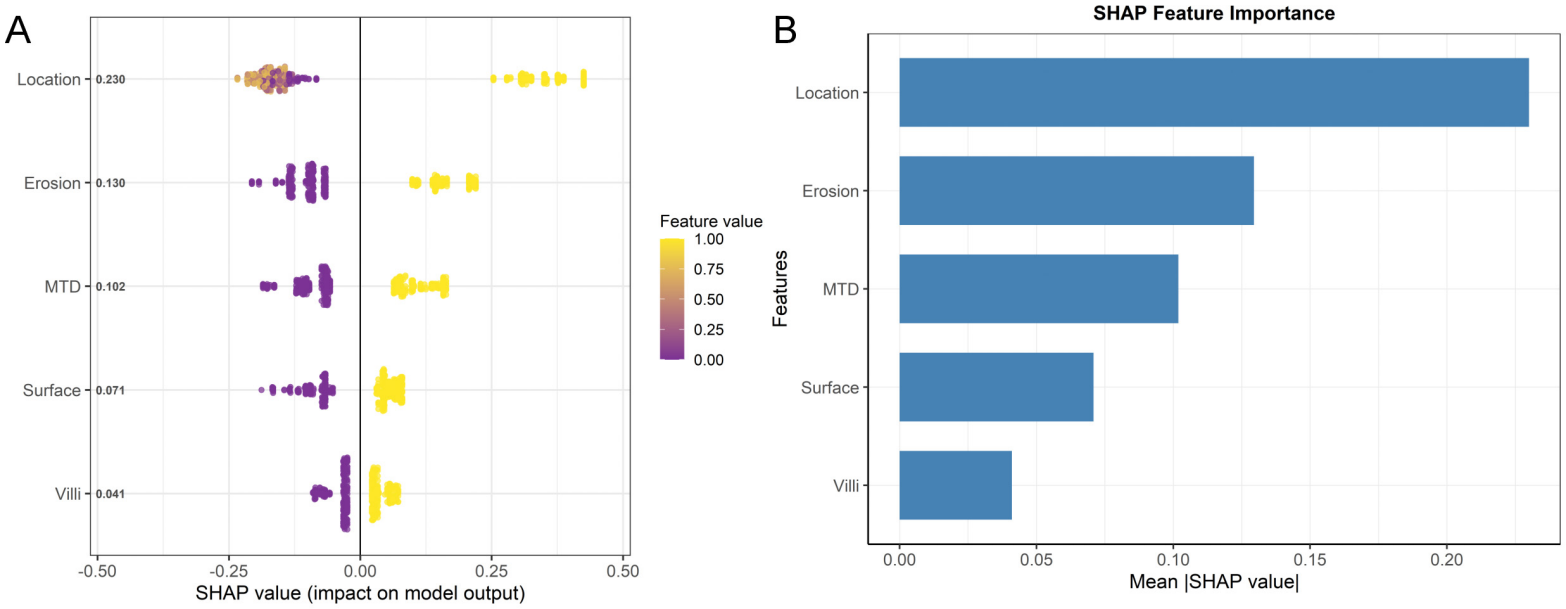
**(A)** SHAP Beeswarm plot. **(B)** SHAP feature importance.

**Figure 6:**
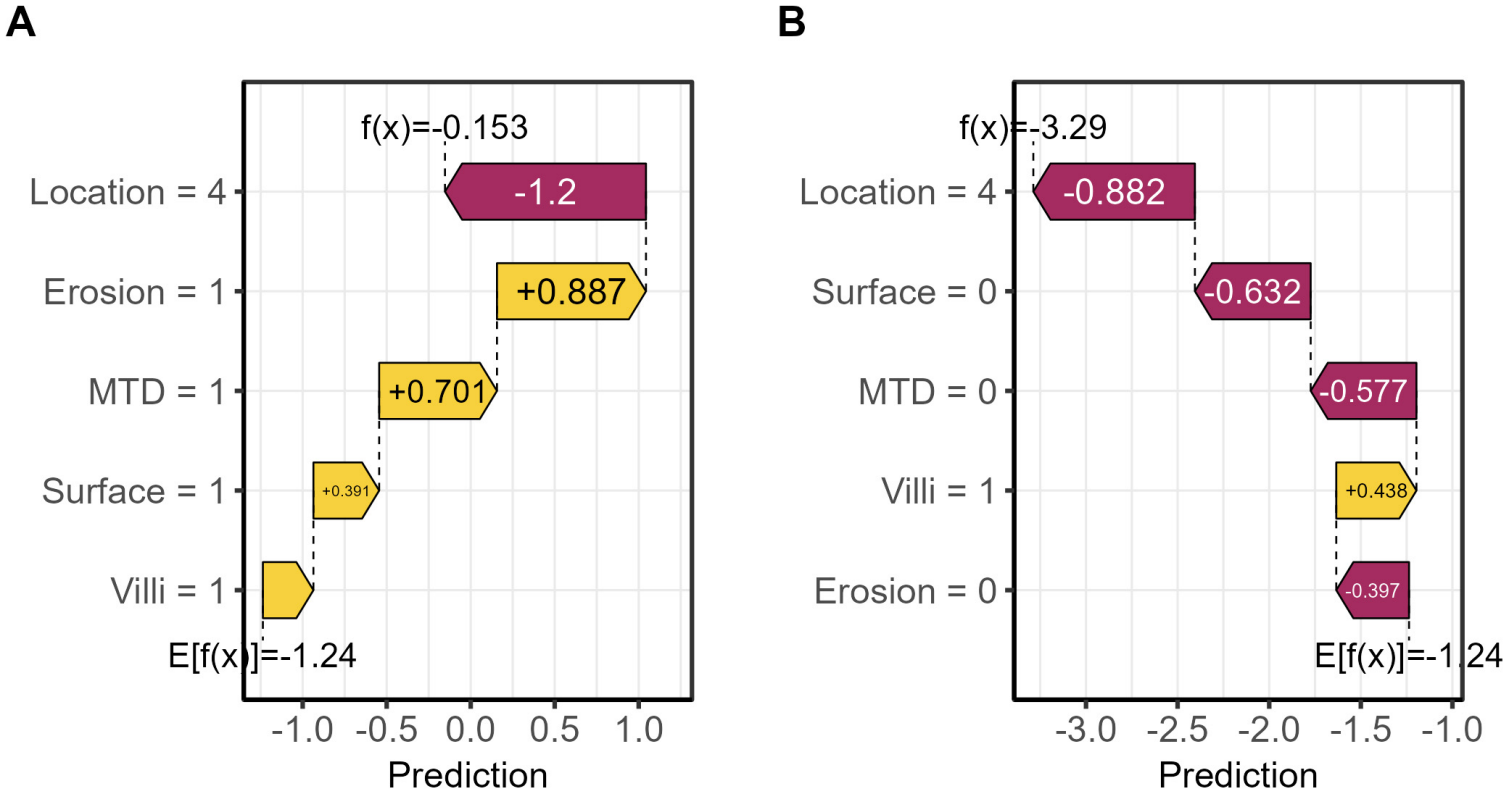
Comparison of SHAP waterfall plots between representative cases. **(A)** Upgraded case. **(B)** Non-upgrade case.

**Figure 7:**
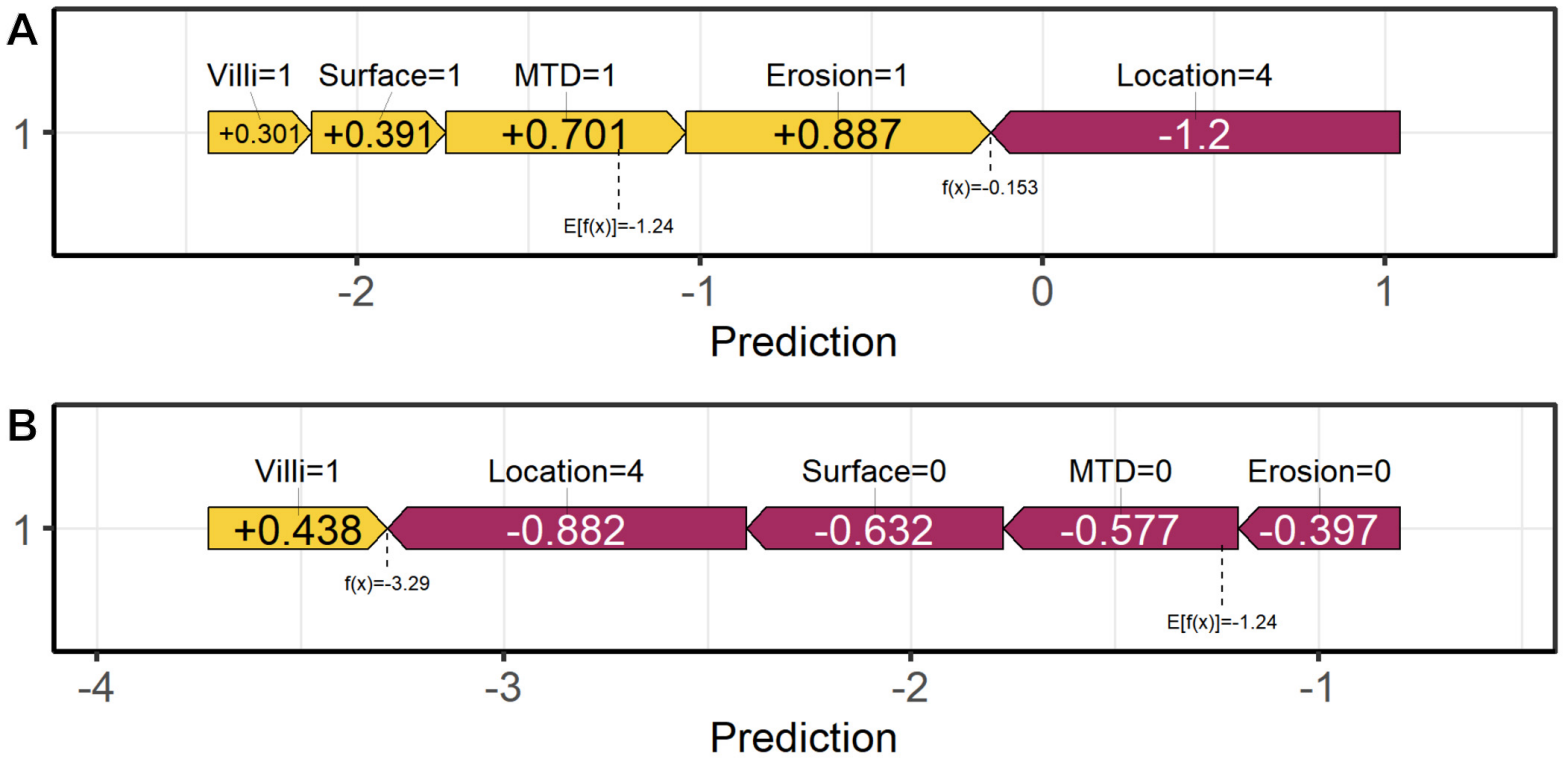
Comparison of SHAP force plots between representative cases. **(A)** Upgraded case. **(B)** Non-upgrade case.

As shown in Figure 5, the distribution of SHAP values across different feature values is presented. The color scale represents feature values, with yellow indicating higher values and purple indicating lower values. Higher feature values are generally associated with a greater contribution to the predicted risk of pathological upgrading. For binary variables such as lesion location, the two categories are displayed at opposite ends of the color spectrum according to their encoded values. This enables consistent visualization across both binary and continuous features. The analysis revealed positive SHAP contributions for features including rectal location, presence of erosion, maximum diameter ≥10 mm, surface redness and roughness, and villous structure. The SHAP feature importance plot identified “Location” as the most influential variable for model prediction. Additionally, SHAP waterfall plots and force plots were generated to explore prediction mechanisms at the individual level. In a representative case with pathological upgrading, multiple high-risk features contributed positively, resulting in a predicted risk substantially above the baseline. Polyp erosion was identified as the primary driving factor. In contrast, for non-upgrade cases, non-rectal location was the key protective factor. These visualizations illustrate the positive and negative contributions of each feature to individual predictions, thereby enhancing model transparency and providing a basis for individualized clinical decision-making (Figures 6, 7).

**Figure 8:**
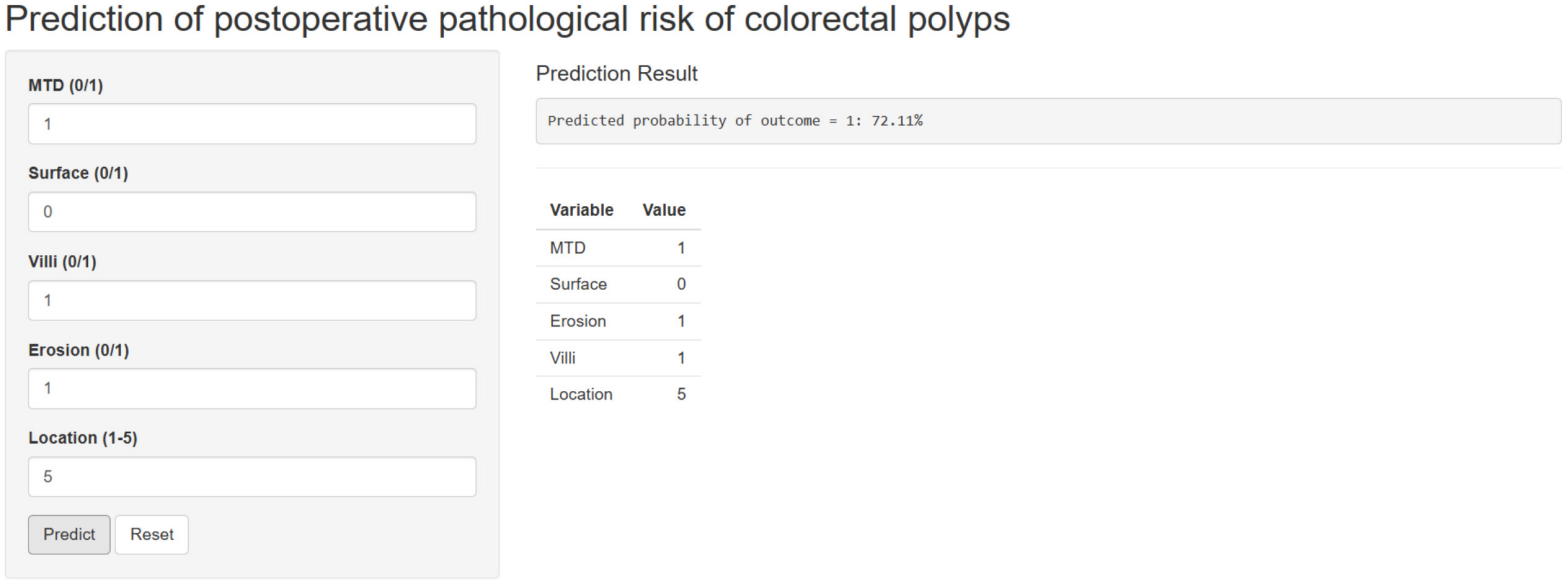
An online web calculator based on the XGBoost model.

Finally, a user-friendly online calculator was developed based on the top-performing XGBoost model (Figure 8). The calculator is available at: https://changchangzhang2001.shinyapps.io/blsj/. It is compatible with standard web browsers, supports keyboard-based interaction, and is designed to be accessible for users with varying levels of technical proficiency. The tool is freely available for clinical and research use without requiring specialized technical expertise. To ensure long-term accessibility, the calculator will remain freely accessible for at least 3 years following publication. The source code can be obtained from the corresponding author upon reasonable request.

## 4 Discussion

Colorectal polyps are widely recognized as key precancerous lesions in the adenoma–carcinoma sequence and represent an important target for colorectal cancer (CRC) prevention (28, 29). Despite the expansion of screening programs, CRC remains a major global public health burden, with substantial impacts on morbidity, mortality, and healthcare resource utilization. Improving the accuracy of risk assessment for colorectal polyps is therefore essential for optimizing patient management and enhancing the efficiency of CRC prevention strategies (3–5). Pathological upgrading, defined as a higher histological grade in resection specimens compared with preoperative biopsy, remains a clinically relevant problem. Misclassification may delay treatment in high-risk patients or lead to unnecessary procedures in low-risk individuals. Accurate preoperative risk stratification is therefore critical.

In this study, a machine learning model for predicting pathological upgrading is developed and validated using routinely available clinical and endoscopic features. Among the six candidate algorithms, XGBoost shows the best overall performance, with the highest discriminative ability in both training and test cohorts and acceptable calibration. The NNet model achieves the lowest Brier score in the test cohort, suggesting better calibration in probability estimation. These findings indicate that machine learning methods can improve risk prediction in clinical settings characterized by heterogeneous and non-linear relationships. The integration of SHAP analysis and the development of an online calculator further enhance model transparency and clinical applicability.

A key issue addressed in this study is the discrepancy between biopsy findings and postoperative pathology. Previous studies have shown that biopsy may fail to capture the most advanced histological component of colorectal lesions, particularly in large or heterogeneous polyps (6–8). This limitation has direct clinical implications because treatment decisions often rely on preoperative assessment. Underestimation may result in insufficient treatment, whereas overestimation may lead to overtreatment. At the population level, such inaccuracies may reduce the efficiency of screening programs. A reliable tool for predicting pathological upgrading may therefore improve pre-treatment decision-making.

The present study identified five key predictors of pathological upgrading: maximum tumor diameter, surface color, erosion, villous structure, and lesion location. These variables were consistently selected by both LASSO regression and the Boruta algorithm, supporting their robustness and relevance. Notably, all predictors are readily obtainable in routine clinical practice, enhancing the feasibility of implementing the model in diverse healthcare settings. Consistent with prior research, the largest tumor diameter was found to be a significant predictor of pathological upgrading, linking larger lesions to a higher likelihood of advanced neoplasia and histological inconsistencies (30). Larger polyps are more likely to exhibit internal heterogeneity, increasing the likelihood that biopsy sampling may miss areas of higher-grade dysplasia or carcinoma. In addition, increased lesion size may reflect prolonged tumor growth and accumulation of genetic alterations. Clinically, this finding underscores the importance of careful evaluation of larger lesions. From a public health perspective, tumor size represents a simple and intuitive indicator that may support risk-based triage and prioritization of patients for advanced endoscopic procedures, particularly in resource-constrained settings. Surface color and erosion were also identified as significant predictors. Changes in mucosal color, such as redness or roughness, may reflect increased vascularity or metabolic activity associated with neoplastic progression (31). Similarly, erosion may indicate disruption of mucosal integrity and more aggressive biological behavior (32). These endoscopic features can be assessed during routine colonoscopy without additional cost or intervention, making them particularly valuable for large-scale application. Their inclusion in the model suggests that visual characteristics observed during endoscopy can provide meaningful information beyond biopsy findings alone. Standardizing the assessment of such features may improve consistency across operators and enhance the accuracy of risk stratification in screening programs.

Villous structure was another important predictor, consistent with its well-established association with advanced histology and malignant potential (6, 33). Lesions with villous components are more likely to harbor high-grade dysplasia and may be more prone to pathological upgrading. The inclusion of this variable enhances the biological plausibility of the model and aligns with established clinical knowledge. In addition, lesion location, particularly rectal location, was identified as the most influential factor in SHAP analysis. The results indicate that differences in anatomy within the colorectum might affect the likelihood of upgrading. Possible explanations include variations in local microenvironment, fecal stasis, exposure to carcinogens, and inflammatory conditions (30, 34). From a public health perspective, identifying high-risk anatomical sites may contribute to more targeted surveillance strategies and improved allocation of endoscopic resources.

An important methodological strength of this study is the use of multiple machine learning algorithms for model development and comparison. Six models were evaluated, allowing selection of the most appropriate approach based on empirical performance. The superior performance of XGBoost is consistent with its ability to capture non-linear relationships and complex feature interactions. Pairwise comparisons using the DeLong test demonstrated that the AUC of the XGBoost model was significantly higher than those of the other models in the test cohort, indicating that its advantage is unlikely to be due to random variation (35). Model performance was evaluated using multiple complementary metrics, including accuracy, sensitivity, specificity, Cohen's kappa, and the Matthews correlation coefficient. Calibration was assessed using the Brier score and calibration curves (18), and DCA was used to evaluate clinical utility across a range of threshold probabilities. Given the moderate class imbalance in the dataset (event rate: 25.3%), no resampling techniques, such as SMOTE or ROSE, and no class weighting strategies were applied. Instead, model performance was assessed using metrics that are more robust to class imbalance, including the Matthews correlation coefficient and the Brier score. During model selection, discriminative ability was prioritized as the primary evaluation criterion because of its central role in distinguishing high-risk from low-risk individuals in risk stratification. Based on this criterion, together with calibration and overall classification performance, XGBoost was selected as the optimal model. Notably, the NNet model showed superior calibration performance, suggesting its potential value in settings that require accurate probability estimation. Therefore, differences in model performance should be interpreted in the context of specific clinical application scenarios.

To further demonstrate the performance advantage of the XGBoost model, we compared its predictive accuracy with that of established risk stratification tools and simpler modeling approaches. The NICE classification, a widely used optical diagnostic system, has shown variable diagnostic accuracy across studies, typically ranging from approximately 60% to 85%, depending on operator expertise and lesion characteristics (36). In comparison, the XGBoost model in the present study achieved more stable and favorable performance, with a test AUC of 0.863 and an accuracy of 81.5%, using only white-light endoscopic features and routinely available clinical variables. When compared with a logistic regression model constructed using the same predictors, XGBoost showed superior discriminative ability, together with higher specificity and MCC, and these differences were statistically significant based on the DeLong test (Table 2). Although XGBoost is more complex than traditional statistical models or rule-based systems such as the NICE classification, this added complexity is justified by its ability to capture non-linear relationships and interactions among predictors, including lesion location, erosion, and villous structure—patterns that are often not fully addressed by simpler approaches (37). Importantly, the integration of SHAP analysis provides transparent, case-level explanations, thereby reducing concerns regarding the “black-box” nature of machine learning models and improving clinical interpretability. With the increasing integration of predictive tools into electronic health record systems, the computational burden of XGBoost is unlikely to represent a practical limitation, supporting its feasibility for real-time risk assessment in screening settings.

Interpretability remains a major challenge for the clinical implementation of machine learning models. This study utilized SHAP analysis to provide both global and individual-level explanations for model predictions, enabling clinicians to better understand the contribution of each feature. The development of an online calculator further enhances the translational value of the model by offering an accessible tool for personalized risk prediction. Such tools may support clinical decision-making and improve consistency in practice.

The results of this research have wider consequences for risk-focused prevention approaches in CRC. As healthcare systems increasingly move toward precision medicine and precision prevention, there is growing emphasis on tailoring interventions based on individual risk profiles. In the context of CRC, this involves not only detecting polyps but also identifying which lesions are most likely to progress and require more aggressive management. The model developed in this study contributes to this approach by enabling preoperative identification of high-risk lesions. This may help reduce both undertreatment of aggressive lesions and overtreatment of low-risk cases, thereby improving the overall efficiency and sustainability of screening programs.

This study has several limitations that should be acknowledged. First, its retrospective single-center design and lack of independent external validation may limit the generalizability and transportability of the findings. Second, potential selection bias exists, as only lesions with complete endoscopic resection and pathological confirmation were included, whereas biopsy-confirmed benign polyps without resection were excluded. This may have led to an underestimation of the true rate of pathological upgrading and may limit applicability to conservatively managed lesions. In addition, the relatively low event rate could affect the stability of model estimates. Third, several predictors were based on endoscopic features subject to operator-related variability, including differences in interpretation and clinical experience. Interobserver variability was not assessed, and reproducibility across operators and centers remains uncertain. Operator-related factors, such as experience and procedural volume, were also not incorporated and may act as unmeasured confounders, affecting model robustness. Finally, some relevant predictors were not included, such as biopsy histological subtypes and advanced endoscopic imaging features. In addition, certain variables were simplified; for example, maximum tumor diameter was dichotomized to improve clinical usability, which may have resulted in some loss of information. Future studies should explore alternative modeling strategies, including multiple cutoff values or continuous representations, to better capture potential non-linear relationships. Our team plans to conduct a multicenter external validation study across 2–3 independent institutions to further assess the transportability of the model prior to clinical implementation.

## 5 Conclusion

Using routinely available clinical and endoscopic features, we developed and compared six machine learning models for predicting pathological upgrading in colorectal polyps. XGBoost showed the strongest overall performance among the candidate algorithms. The proposed model may assist in preoperative risk assessment, support individualized endoscopic management, and facilitate earlier identification of lesions at higher risk of pathological upgrading.

## Data Availability

The raw data supporting the conclusions of this article will be made available by the authors, without undue reservation.
