# Supplementary material for: Preoperative risk stratification for pathological upgrading in colorectal polyps using explainable machine learning: implications for screening optimization and resource allocation
Source: Front Public Health. 2026 May 21;14:1845071. doi: 10.3389/fpubh.2026.1845071 (PMC13233670; doi:10.3389/fpubh.2026.1845071)
Supplement: Supplementary file 1 [file Supplementary_file_1.docx]

Supplementary Material

# Supplementary Figures and Tables

## Supplementary Table 1

**Table S1**. **Proportion of missing data for each variable prior to multiple imputation**

| Variable | Missing(n) | Missing(%) |
| --- | --- | --- |
| Gender | 0 | 0% |
| Age | 0 | 0% |
| Smoking history | 0 | 0% |
| BMI | 16 | 2.70% |
| CEA | 11 | 1.85% |
| Family history of CRC | 0 | 0% |
| CMS | 0 | 0% |
| Maximum tumor diameter | 0 | 0% |
| Pedunculated tumor | 0 | 0% |
| Number of biopsy blocks | 0 | 0% |
| Villous | 0 | 0% |
| Surface | 0 | 0% |
| Erosion | 31 | 5.23% |
| Number of tumor | 0 | 0% |
| Intestinal cleanliness | 0 | 0% |
| Location | 0 | 0% |

**Table S2.Baseline characteristics of the training and test cohorts**

| Variables | Train set(n=416) | Test set(n=177) | P-value |
| --- | --- | --- | --- |
| Gender[n(%)] |  |  | 0.641 |
| Female | 58(13.9%) | 28(15.8%) |  |
| Man | 358(86.1%) | 149(84.2%) |  |
| Age[M(Q1-Q3,years] | 58(51-72) | 56(50-69) | 0.176 |
| Smoking history[n(%)] |  |  | 0.790 |
| No | 79(19.0%) | 36(20.3%) |  |
| Yes | 337(81.0%) | 141(79.7%) |  |
| BMI[n(%)] |  |  | 0.966 |
| 24-28 kg/m² | 210(50.5%) | 89(50.3%) |  |
| ＜24 kg/m² | 132(31.7%) | 55(31.1%) |  |
| ＞28 kg/m² | 74(17.8%) | 33(18.6%) |  |
| CEA[n(%)] |  |  | 0.491 |
| <4.7 μg/L | 363(87.3%) | 150(84.7%) |  |
| ≥4.7 μg/L | 53(12.7%) | 27(15.3%) |  |
| Family history of CRC[n(%)] |  |  | 0.847 |
| No | 377(90.6%) | 162(91.5%) |  |
| Yes | 39(9.4%) | 15(8.5%) |  |
| CMS[n(%)] |  |  | 0.340 |
| No | 268(64.4%) | 106(59.9%) |  |
| Yes | 148(35.6%) | 71(40.1%) |  |
| Maximum tumor diameter[n(%)] |  |  | 0.080 |
| ＜10mm | 222(53.4%) | 109(61.6%) |  |
| ≥ 10mm | 194(46.6%) | 68(38.4%) |  |
| Pedunculated tumor[n(%)] |  |  | 0.515 |
| No | 165(39.7%) | 76(42.9%) |  |
| Yes | 251(60.3%) | 101(57.1%) |  |
| Number of biopsy blocks[n(%)] |  |  | 0.538 |
| 1 piece | 342(82.2%) | 141(79.7%) |  |
| ≥ 2pieces | 74(17.8%) | 36(20.3%) |  |
| Villous |  |  | 0.194 |
| No | 221(53.1%) | 83(46.9%) |  |
| Yes | 195(46.9%) | 94(53.1%) |  |
| Surface[n(%)] |  |  | 0.081 |
| Normal mucosal color | 159(38.2%) | 82(46.3%) |  |
| red | 257(61.8%) | 95(53.7%) |  |
| Erosion[n(%)] |  |  | 0.632 |
| No | 241(57.9%) | 107(60.5%) |  |
| Yes | 175(42.1%) | 70(39.5%) |  |
| Number of tumor[n(%)] |  |  | 0.104 |
| Single | 302(72.6%) | 116(65.5%) |  |
| Multiple | 114(27.4%) | 61(34.5%) |  |
| Intestinal cleanliness[n(%)] |  |  | 1.000 |
| Adequate Bowel Preparation | 394(94.7%) | 168(94.9%) |  |
| Inadequate Bowel Preparation | 22(5.3%) | 9(5.1%) |  |
| Location[n(%)] |  |  | 0.362 |
| Ascending colon | 48(11.5%) | 25(14.1%) |  |
| Transverse colon | 48(11.5%) | 24(13.6%) |  |
| Descending colon | 70(16.8%) | 33(18.6%) |  |
| Sigmoid colon | 109(26.2%) | 33(18.6%) |  |
| Rectum | 141(33.9%) | 62(35.0%) |  |

**TableS3.Pairwise comparisons of AUCs between the XGBoost model and other models in the test set using the DeLong test**

| Model (Comparison) | AUC (XGBoost) | AUC (Comparator) | AUC Difference | P value |
| --- | --- | --- | --- | --- |
| RF | 0.863 | 0.735 | 0.127 | <0.001 |
| CART | 0.863 | 0.793 | 0.070 | 0.004 |
| NNet | 0.863 | 0.784 | 0.078 | 0.012 |
| LR | 0.863 | 0.693 | 0.170 | <0.001 |
| GBM | 0.863 | 0.794 | 0.069 | 0.005 |

**1.2 Supplementary Figure 1**


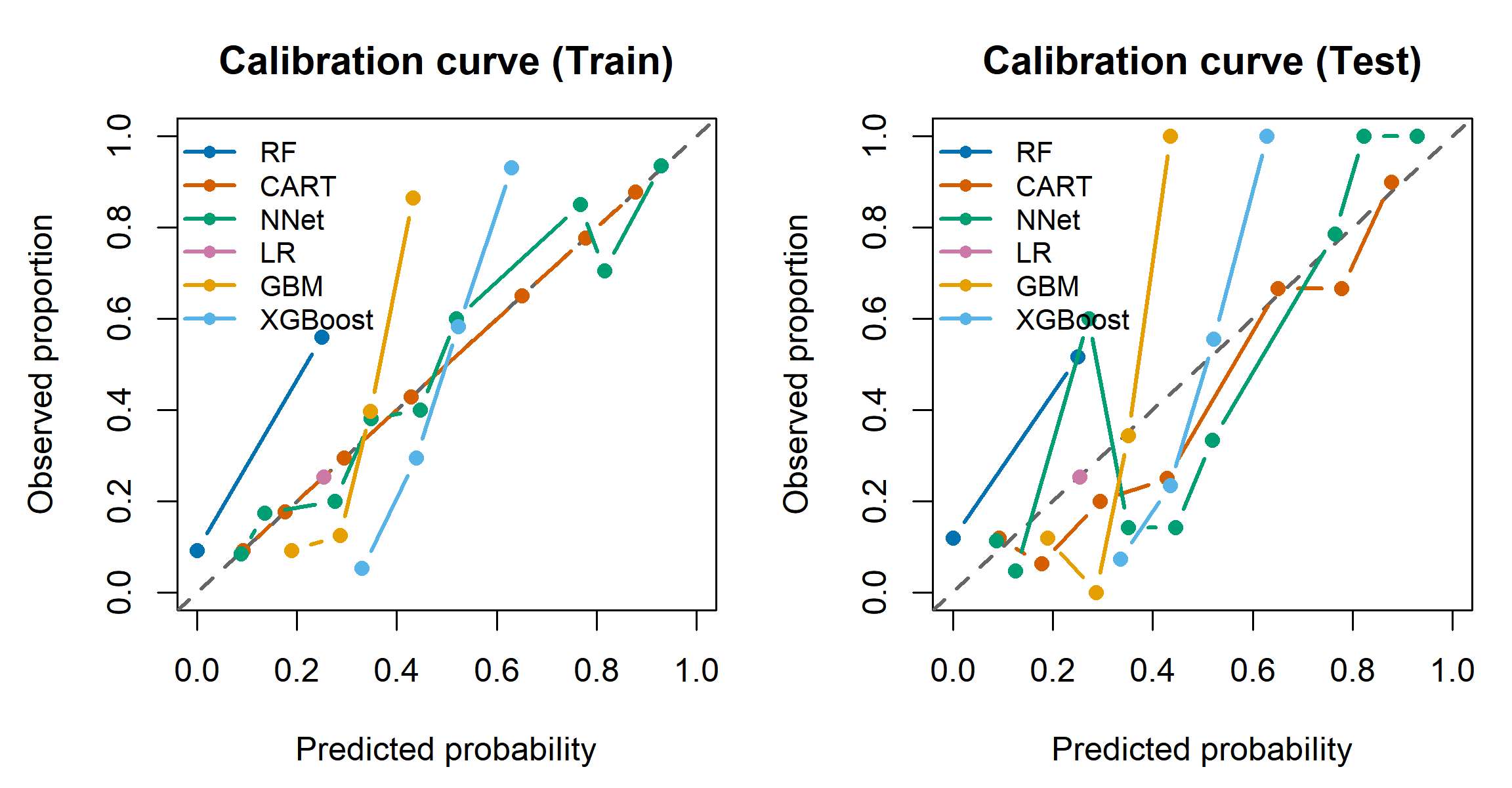


Figure S1.Calibration curves for each model in the training and test sets. The diagonal dashed line represents perfect calibration, where the predicted probability equals the observed proportion. Points closer to the diagonal line indicate better agreement between model predictions and actual outcomes.
